# Spontaneous perforation of the cystic duct in streptococcal toxic shock syndrome: a case report

**DOI:** 10.1186/1752-1947-2-338

**Published:** 2008-10-29

**Authors:** Henrik Endeman, David A Ligtenstein, Heleen M Oudemans-van Straaten

**Affiliations:** 1 Onze Lieve Vrouwe Gasthuis, Department of Intensive Care Medicine, Oosterpark 9, PB 95500, 1090 HM Amsterdam, the Netherlands; 2 Ruwaard van Putten Ziekenhuis, Department of Surgery, PB 777, 3200 GA Spijkenisse, the Netherlands

## Abstract

**Introduction:**

Streptococcal toxic shock syndrome is a complication of group A streptococcal infection, most often originating from the skin. The syndrome is characterized by fever, hypotension and multiple organ failure. Mortality rate may be as high as 80%.

**Case presentation:**

A 25-year-old man of Indian origin presented with abdominal complaints, rash and fever after an episode of pharyngitis. The patient was operated and a biliary peritonitis was found caused by perforation of the cystic duct in the absence of calculi. Cholecystectomy was performed, but after the operation, the patient's condition worsened and multi-organ failure developed. Group A streptococci were cultured in blood taken at admission and streptococcal toxic shock syndrome was diagnosed. Treatment consisted of antibiotics, corticosteroids, immunoglobulin and supportive treatment for haemodynamic, respiratory and renal failure.

**Conclusion:**

This is a patient with streptococcal toxic shock syndrome complicated by spontaneous perforation of the cystic duct. Spontaneous perforation of the cystic duct is a rare finding, most often reported in children and secondary to anatomic defects. We found only one similar adult case in the literature. Perforation may be due to microthrombosis and ischaemia, and so be a part of the multi-organ failure often found in streptococcal toxic shock syndrome.

## Introduction

Streptococcal toxic shock syndrome (StrepTSS) is caused by beta-haemolytic streptococcus group A (M-1 strain) most frequently originating from an infection of the skin (cellulitis or erysipelas), pharynx or vagina [1,2]. StrepTSS is defined as 1) isolation of streptococcus group A, 2) hypotension and two of the following signs: renal impairment (acute renal failure, ARF), coagulopathy (diffuse intravascular coagulation, DIC), liver involvement, adult respiratory distress syndrome (ARDS), erythematous macular rash or soft tissue necrosis [1]. StrepTSS is reported in three age groups: children (0 to 15 years), young adults (24 to 44) and elderly (65+). StrepTSS in adults is associated with alcohol abuse, corticosteroid use, diabetes mellitus, heart and lung diseases, HIV/AIDS, malignancy, peripheral vascular disease, recent varicella/influenza infection and living in a nursing home [1,2]. Mortality of StrepTSS is 33% up to 81% [1]. Other infections associated with StrepTSS are cerebral empyema, endocarditis, endophthalmitis, lymphangitis, mediastinitis, meningitis, myositis, necrotizing fasciitis, osteomyelitis, pelvic infection, peritonitis, puerperal and postpartum infections, septic arthritis, thrombophlebitis (intravenous drug abuse), upper and lower respiratory tract infections (including otitis media) and urinary tract infection [1]. The classic clinical picture of StrepTSS is an acute febrile illness, beginning with mild viral symptoms and involves a minor soft tissue infection or upper airway infection that progresses to shock, multi-organ failure (MOF) and death [1,2]. An initial viral infection causes damage to the mucosa, thus facilitating penetration of group A streptococcus.

In this case report, we present a patient with StrepTSS with a rare complication: spontaneous perforation of the cystic duct.

## Case presentation

A 25-year-old formerly healthy Hindu man, living in the Netherlands from birth, was admitted to our Intensive Care Unit (ICU) after abdominal surgery in a hospital outside our region. The patient presented in that hospital one day before operation with fever and moderate abdominal complaints. One week before, he became ill with fever, sore throat and red-yellow macular discoloration on his extremities and thorax. The week before, his girlfriend, an employee of a kindergarten, had similar symptoms, but she recovered. After a few days, his fever and sore throat disappeared, but then he developed a second phase of fever, accompanied by nausea, vomitus (once), dark coloured urine and a single passage of watery, possibly discoloured, stool.

At presentation in the hospital, the patient had fever (40°C) and tachycardia (150/minute). On clinical examination, the patient had diffuse abdominal tenderness. Skin lesions had resolved. Laboratory examination revealed signs of inflammation (C-reactive protein (C-RP), 294 mg/litre; white blood cells (WBC), 3.8 × 10^9^/litre; 50% rods) and cholestasis (total bilirubin, 100 μmol/litre; conjugate bilirubin, 63 μmol/litre; alkaline phosphatase (AF), 168 U/litre and gamma glutamyl transferase (γGT), 241 U/litre). Ultrasound and computed tomography (CT) scan of his abdomen showed no abnormalities, especially no signs of cholecystitis or cholangitis (including the absence of cholecysto- and choledocholithiasis). Laparotomy was performed because of progressive abdominal complaints in combination with shock, and revealed a biliary peritonitis due to a pinpoint perforation of the base of the cystic duct. Gallbladder and common bile duct were free of stones, but the cystic duct looked inflamed and necrotic. Peritoneal lavage and cholecystectomy were performed. Postoperative course was complicated by severe septic shock with MOF including ARDS, ARF and DIC. In cultures of blood taken on admission, a beta-haemolytic streptococcus group A was isolated.

The patient was transported to our ICU with refractory hypotension despite high-dosage noradrenalin, pulmonary insufficiency requiring high-pressure ventilation (positive end expiratory pressure (PEEP), 20 mmH_2_O; FiO_2 _70%) and oliguria. Clinical and laboratory parameters at admission to our ICU are shown in Table 1. Chest X-ray showed bilateral patchy infiltrates without cardiac enlargement. The patient was diagnosed as suffering from StrepTSS with MOF complicated by spontaneous perforation of the cystic duct and biliary peritonitis.

**Table 1:** Laboratory results at admission after transfer to ICU

Parameter	Results
Haemoglobin	6.4 mmol/litre
White blood cell count (WBC)	10.6 × 10^3^/litre (59% rods)
Platelets	68 × 10^3^/litre
C-reactive protein	149 mg/litre
PTT	16.8 seconds
APTT	41.6 seconds
Antithrombin III	35 g/litre
Fibrinogen	2 g/litre
d-dimer	45,700 mcg/litre
pH	7.22
pCO_2_	50 mmHg
pO_2_	74 mmHg
Bicarbonate	20.2 mmol/litre
Sodium	140 mmol/litre
Potassium	4 mmol/litre
Chloride	107 mmol/litre
Urea	12.9 mmol/litre
Creatinine	258 μmol/litre
Albumin	20 g/litre
Total/conjugated bilirubin	74/67 μmol/litre
Alkaline phosphatase	97 U/litre
γGT	91 U/litre
ALAT	152 U/litre
ASAT	317 U/litre
Creatine kinase	2563 mmol/litre

Treatment consisted of our standard pre-emptive antibiotics for abdominal sepsis (cefotaxime 1 g four times daily, initially combined with ciprofloxacin and metronidazol) in combination with corticosteroids and immunoglobulin (30 g intravenous immunoglobulin daily for 5 consecutive days). After blood cultures were positive for streptococcus group A, ciprofloxacin and metronidazol were stopped. Supportive therapy consisted of mechanical ventilation (initially in the prone position), fluid resuscitation in combination with inodilators (enoximone), vasodilators (nitroglycerin) and vasoconstrictors (high-dose dopamine and a short period of noradrenalin), selenium and selective decontamination of the digestive tract. Cefotaxime was continued because the patient's condition and inflammatory markers improved. On day 5, he was successfully weaned from mechanical ventilation. At this time, his platelet count had recovered and renal function was improving. Renal replacement therapy was not necessary. After 7 days of treatment in our ICU, he returned to a hospital in his home region. His close relatives were advised to take a prophylactic macrolide for 5 days. Pathologic examination of the gallbladder showed acute inflammation without bacteria and without stones.

## Discussion

This patient was diagnosed as suffering from StrepTSS originating from an upper respiratory infection, either viral or streptococcal pharyngitis, and fulfilled the diagnostic criteria for StrepTSS (isolation of streptococcus group A, hypotension, ARDS, renal insufficiency, DIC). The streptococcus group A likely originated from his girlfriend who worked in a kindergarten. Apart from a possible viral infection, our patient had no evident risk factors. His clinical features were classical: acute febrile illness, beginning with mild viral symptoms originating from the upper airways with progression to MOF.

Spontaneous perforation of the cystic and/or common bile duct as a complication of StrepTSS has not been reported before in adults. Perforation of the intra- or extrahepatic biliary tract is rare. In adults, most cases of non-traumatic perforation of the biliary tract are due to obstruction by stones (or tumours) resulting in increased ductal pressure, cholangitis and eventually necrosis and perforation [3,4]. There are a few reports of adult patients with spontaneous perforation in the absence of calculi and only one of perforation of the cystic duct as in our patient. In this patient, perforation of the cystic duct was due to acalculous cholecystitis [5]. Clinical features of nontraumatic perforation of the bile ducts in adults are acute abdominal pain and febrile illness, sometimes in combination with elevated bilirubin, especially in the case of stones [4]. All three features were present in our patient, though he did not suffer from biliary stone disease. The elevated bilirubin in our patient was due to hepatic insufficiency as part of the multi-organ dysfunction syndrome. CT scan or ultrasonography may show non-specific findings such as (perihepatic) fluid and, in the case of stones, obstructive lesions in the biliary tract [4]. The combination of biliary stone disease, acute abdominal complaints and increased inflammatory parameters is an indication for the presence of nontraumatic perforation of the biliary tract, especially in combination with perihepatic fluid on radiological examination of the abdomen. In the absence of stones, definitive diagnosis can only be made by laparotomy.

Spontaneous perforation of the biliary tract in the absence of gallstones is mostly reported in (young) children. Mechanisms of perforation of the biliary tract are biliary tract anomalies (especially cysts), ascariasis and cholecystitis [6-8]. A possible mechanism of spontaneous perforation of the cystic duct in our patient is local necrosis due to microcirculatory failure as a result of hypoperfusion and microthrombosis. This resembles the case reported by Shah and Webber where spontaneous perforation of the common bile duct was due to acalculous cholecystitis, which is probably also caused by diminished local microcirculation [5]. Most cases of spontaneous perforation of the biliary tract in childhood are reported in children of African or Asian ethnicity; our patient was of Indian origin. The pathophysiological role of ethnicity is unknown. Treatment of spontaneous perforation of the biliary tract consists of cholecystectomy and, in the case of obstruction, external or internal drainage of the biliary tract.

Management of StrepTSS consists of treatment of the location of infection (for example, debridement of infected soft tissue), antibiotics and support of failing organ functions. Definitive studies establishing the most effective antibiotic for StrepTSS are not available. Penicillin and clindamycin are the classical choice [1,2]. We applied selective decontamination of the digestive tract to prevent secondary infectious complications, especially ventilator associated pneumonia [9,10]. The systemic part of this strategy consisted of cefotaxime, which also has streptococcal coverage. We preferred treatment with cefotaxime over penicillin and clindamycin, because the latter two also eradicate non-pathogenic endogenous anaerobic bacteria, thereby facilitating acquisition of non-endogenous Gram-negative bacteria or *Clostridium difficile *[11]. Ciprofloxacin and metronidazole, initiated for abdominal sepsis with unknown cause, were discontinued as soon as cultures were present. Ciprofloxacin has no direct killing effects on anaerobes and metronidazole is rapidly inactivated in faeces.

Our haemodynamic support not only focused on restoration of pressure, but additionally of flow in the systemic microcirculation using fluids, inodilatation and vasodilation with enoximone and nitroglycerin [12,13]. The patient would have been eligible for treatment with activated protein C, but his recent operation was a contraindication for activated protein C. Further treatment consisted of corticosteroids [14], selenium [15] and immunoglobulin. Immune-modulation using intravenous immunoglobulin is recognized as a therapy with potential benefits in StrepTSS. Possible effects of intravenous immunoglobulin consist of enhancing phagocytosis, neutralization of toxic mediated effects and induction of regulatory cytokines resulting in suppression of the pro-inflammatory response [16]. This combined anti-inflammatory strategy may be crucial to enhance recovery if hospital acquired infectious complications are under control with selective decontamination of the digestive tract.

## Conclusion

StrepTSS is a severe infectious disease characterized by high mortality and MOF. Perforation of the cystic duct is a rare complication of StrepTSS. Perforation of the cystic duct is possibly caused by alteration in the local microcirculation leading to necrosis and eventually perforation.

## Abbreviations

AF: alkaline phosphatase; ALAT: alanine aminotransferase; APTT: activated partial thromboplastin time; ARDS: adult respiratory distress syndrome; ARF: acute renal failure; ASAT: aspartate aminotransferase; C-RP: C-reactive protein; CT: computed tomography; DIC: disseminated intravascular coagulation; γGT: gamma glutamyl transferase; ICU: Intensive Care Unit; MOF: multi organ failure; PEEP: positive end expiratory pressure; PTT: partial thromboplastin time; StrepTSS: streptococcal toxic shock syndrome; WBC: white blood cells

## Consent

Written informed consent was obtained from the patient for publication of this case report and any accompanying images. A copy of the written consent is available for review by the Editor-in-Chief of this journal.

## Competing interests

The authors declare that they have no competing interests.

## Authors' contributions

The patient was initially treated by DAL and sent to the ICU where treatment was taken over by HE and HMO. The case-report was written by HE and extensively reviewed by HMO. Results of the operation and pathological examination were added by DAL.
